# Is MDMX the better target?

**DOI:** 10.18632/aging.101479

**Published:** 2018-06-13

**Authors:** Ning Kon, Wei Gu

**Affiliations:** 1 Institute for Cancer Genetics, College of Physicians and Surgeons of Columbia University, New York, NY 10032, USA; 2 Department of Pathology and Cell Biology, College of Physicians and Surgeons of Columbia University, New York, NY 10032, USA

**Keywords:** p53 3KR mutant, mdm2 KO, mdmx KO

The functions of tumor suppressor p53 are much appreciated in combating tumorigenesis when cells encounter genotoxic and other deleterious stresses. Under those circumstances, p53 stabilities and transcriptional activities increase dramatically and often lead to activation of genes and trigger pathways that cause terminal and irreversible fate for the damaged cells. Meanwhile, the p53 functions in metabolic regulation have gained attractions for their roles in normal conditions and in tumor suppression. To dissect the physiological role of acetylation in modulating p53 functions, we previously established a *p53-3KR* mutant mice where the acetylation defective p53 mutant losses its ability to activate apoptosis, senescence and growth arrest [1]. Importantly, the *p53-3KR* mice do not succumb to early onset tumor formation compared to *p53* knockout mice, which allow us to uncover p53 activities beyond those canonical tumor suppression functions of p53. To further enhance the remaining functions of p53, *p53-3KR* mutant mice are crossed with *mdm2* or *mdmx* knockout mice individually, to study the stability and transcriptional activation of p53-3KR mutant in the absence of either MDM2 or MDMX.

**Figure 1:**
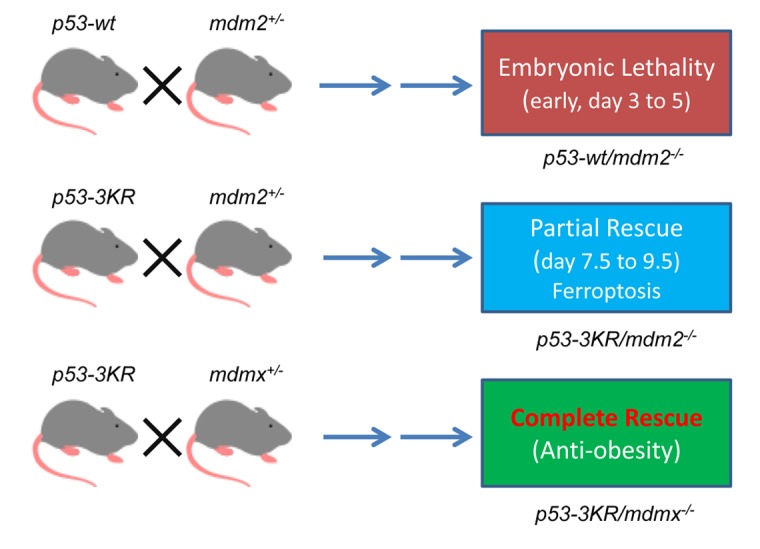
Differential effects of p53 function by inactivation of *mdm2* or *mdmx* by using mouse models.

Although *p53-3KR* mutant only partially rescued the lethality of *mdm2* knockout mice, p53-3KR protein levels dramatically increased in the absence of MDM2, demonstrating again that MDM2 is critical for p53 stability (Figure 1). Since p53-3KR mutant is unable to induce apoptosis, senescence and cell growth arrest, the embryonic lethality in *p53-3KR/mdm2^-/-^* mice led us to discovery of the p53 mediated downregulation of SLC7A11 and subsequent activation of ferroptosis, a form of cell death in response to ROS stress [2]. In contrast, *p53-3KR* mutant was able to completely rescue the lethality of *Mdmx* knockout mice (Figure 1), suggesting the ferroptotic functions of p53-3KR are not activated in the absence of MDMX. Interestingly, there was a modest increase of p53-3KR levels in the absence of MDMX, indicating MDMX only has a limited role in regulating p53 stability, revealing that the Mdm2/Mdmx heterodimer is not absolutely required for p53 degradation [3]. Nevertheless, a number of genes were activated and resulted in metabolic phenotypes. The *p53-3KR/mdmx^-/-^* mice became skinny even though they had no obvious developmental defects. The analysis of these mice revealed that p53 activities were enhanced in *p53-3KR/mdmx^-/-^* mice, which resulted in resistance to fat accumulation in adipose tissues upon high fat diet in these mice [3]. These anti-obesity phenotypes in *p53-3KR/mdmx^-/-^* mice were caused in part by modulation of lipid metabolism and thermogenic programs in adipose tissues. These results elucidate unknown beneficial effects of the p53/MDMX axis in adipose tissue remodeling, and revealed a surprising role of MDMX inhibition in anti-obesity effects beyond, commonly expected, tumor suppression.

Restoration of p53 activity remains an important goal in the quest for more effective cancer therapeutics. Indeed, small molecule inhibitors of MDM2 such as Nutlin-3, are able to activate p53, and exhibits antitumor efficacy in cancer cells that express wild-type p53. Nevertheless, the usage of MDM2 inhibitors in clinics has significant limitations with high toxicity in normal cells [4]. Based on our study, loss of MDMX induces p53 activation but apparently has much less destructive effects than MDM2 inactivation *in vivo*. Thus, it is very likely that targeting MDMX alone might be the better approach in cancer therapy since much less toxicity was observed upon MDMX inactivation. Moreover, specific inhibitors of MDMX may have both tumor suppression and anti-obesity effects.
